# Effects of Quince Gel and Hesperidin Mixture on Experimental Endometriosis

**DOI:** 10.3390/molecules28165945

**Published:** 2023-08-08

**Authors:** Işılay Sezen Ermiş, Engin Deveci, Fırat Aşır

**Affiliations:** 1 Department of Gynecology and Obstetrics, Medical Faculty, Harran University, Şanlıurfa 63050, Turkey; 2 Department of Histology and Embryology, Medical Faculty, Dicle University, Diyarbakır 21280, Turkey; engindeveci64@gmail.com (E.D.); firatasir@gmail.com (F.A.)

**Keywords:** histochemistry, morphometry, TAS, TOS, ultrastructural

## Abstract

Objectives: Endometriosis (EM) is the presence of endometrial tissue outside the uterus. This study aimed to examine the effects of quince gel and hesperidin treatment on uterine tissue in an experimental endometriosis model. Materials and Methods: Thirty-two rats were categorized into four groups as sham, EM, EM+quince gel (QG), and EM+QG+Hesperidin (HES). The endometriosis (EM) model was induced with surgical intervention. Estradiol benzoate (EB) was used to induce endometrial hyperplasia. In the EM group, EB was given to rats for 7 days. The EM+QG group received 2 cc QG for 21 days. HES treatment was given for 21 days after EM induction. At the end of the experiment, blood was taken from the animals and the serum total antioxidant status (TAS) and total oxidant status (TOS) values were studied. Uterine tissues were dissected and processed for histological paraffin embedding. Tissues were fixed in 4% glutaraldehyde solution and processed for ultrastructural analysis. Results: After EM, QG and HES treatment significantly increased the TAS and decreased the TOS value. EM caused epithelial and glandular degeneration, thinning of the basal membranes, and vascular dilatation with increased fibrosis and edema. QG+HES restored the pathology and showed protective effects in uterine tissues. Caspase-3 expression was increased in the epithelium, glands, and muscle layers of the EM group. In EM+QG+HES, hesperidin protected cell survival and decreased Caspase-3 expression in uterine tissues. TNF-α expression was intense in inflammatory cells and the muscle layer in the EM group. HES reduced inflammation by decreasing the TNF-α expression. MAPK expression was increased after EM induction in epithelial, glandular, and inflammatory cells in the EM group. After HES treatment, MAPK expression was mainly negative in cells of uterine tissue in the EM+QG+HES group. Ultrastructurally, in the EM group, organelles were disrupted and dilated and degenerated after EM induction. QG and HES treatment improved cellular organelles. Conclusion: Local vaginal applications can be an alternative treatment method in the endometriosis model via QG+HES treatment promoting cell proliferation and angiogenesis and preventing cell death.

## 1. Introduction

Endometriosis (EM) is defined as the presence of tissue of the endometrium in any part of the body outside of the uterine cavity. The term was first introduced in the 1800s, but scientifically defined in 1927 [1,2]. Several theories have been proposed for the etiology of endometriosis. According to the implantation theory, EM is developed due to regression of tissues in the endometrium towards the tuba uterine, peritoneum and ovaries. Coelomic metaplasia theory states that the parietal peritoneum originating from the coelomic epithelium is pluripotent and metastasizes to the pleura, the mucosa of the genital tract, and the ovaries, leading to induction of EM. According to lymphatic and vascular theory, cells in the endometrium is transported to other organs via vascular structures. An unknown stimulus causes differentiation of peritoneal cells into endometrial cells, causing EM, the induction theory says [3,4,5]. Although the pathophysiology of the endometriosis remains unclear, it is known that endometriosis is a condition dependent on estrogen and resistant to progesterone hormones. The level of estrogen is increased in endometriosis and causes inflammation and growth of endometrium that contributes to symptoms of it. Low levels of progesterone are also thought to play a role in endometriosis. 17β-estradiol (E2) is a key hormone that is associated with endometriosis, inflammation and its symptoms. This local estrogen hormone is mainly produced in endometrial tissues and binds and activates estrogen receptors [6,7]. The clinical presentation of EM is complaints of pain, dysmenorrhea, dysuria, chronic pain in the pelvis, abnormal hemorrhage, and infertility. Hormonal contraceptives, gonadotropin-releasing hormone agonists and antagonists, progestin therapy and aromatase inhibitors are used in the treatment of endometriosis [6,7].

Recently, therapeutic approaches have been developed for the treatment of endometriosis. Medicinal plants have been used for centuries in the treatment of various diseases. Hesperidin is a bioflavonoid glycoside found mostly in citrus fruits [8,9]. From its biochemical structure, the number and specific position of hydroxyl groups in the flavanone aromatic rings show that this active substance has many biological activities. Various studies have reported that hesperidin has anticancer, neuroprotective, antitumor, antioxidant, anti-inflammatory, hypocholesterolemia and hypoglycemic effects [10,11,12].

Quince, or to use its scientific name *Cydonia oblonga* Miller, is a leafy tree belonging to the Rosaceae family. It usually grows in the Middle East, South Africa and Central Europe. It is used as a medicinal plant in the treatment of various effects. Various components of the quince plant are used as sedatives, antipyretics and antidiarrheals [13,14]. Najman et al. [15] showed that quince products are rich in bioactive materials such as tannins, carotenoids, flavonoids, phenolic acids, and total polyphenols. A study revealed that quince includes vitamins (retinol, thiamine, carotene, riboflavin, ascorbic acid) and minerals (potassium, sodium, calcium, iron, magnesium, etc) [16,17]. The seeds include polyphenols (C-glycosil flavones, lucenin-2, vicenin-2, stellarin-2, isoschaftoside, schaftoside, 6-C-pentosyl-8-C-glucosyl chrysoeriol and 6-C-glucosyl-8C-pentosyl chrysoeriol, and kaempferol-3-Orutinoside) [18], fat soluble compounds (tocopherols, phytosterols and phenolic acids) [19], and organic acids (citric, ascorbic, malic, quinic, shikimic, fumaric acids, ursolic acid, tormentic acid, and β-daucosterol) [20]. In an experimental wound healing, 10–20% concentrations of quince seeds mucilage were applied to wound area for 13 days and resulted in complete healing [21]. Alizadeh et al. [22] investigated effects of quince seed extract on the wound infected with Staphylococcus aureus. They showed extracts were found effective against infected wounds.

Quince seed mucilage is a mixture of water-soluble polysaccharides and cellulose. A reverse reaction of esterification (acidic hydrolysis) led to the presence of sugar found in quince seed (D-xylose, 4-Omethyl glucose, and D-glucose). Quince seed mucilage is formed by soaking or boiling the quince seed. In addition to the wound-healing effect, the mucilage is used via the route of the throat for bronchitis and laxative properties [21,23]. Wang et al. [24] studied the antioxidant activity of Chinese quince and found that quince provides the essential role of quince seed oil processing, particularly as an ingredient in functional foods and medicines. Mirzaii et al. [25] stated that quince seed mucilage has antifungal activity in their study on herbal nano hydro gel synthesis.

The bioactivity of quince gel (QG) and hesperidin (HES) seems to be numerous. Since HES contains sugar, a combination of HES with QG may be more effective in biological activities. Moreover, hesperidin is commercially sold under different brand names as a supplement. This study investigates the impacts of quince gel and hesperidin on the uterus tissue in an experimental endometriosis model.

## 2. Results

### 2.1. Biochemical Findings

The total antioxidant status (TAS) and total oxidant status (TOS) values in sham, endometriosis (EM), EM+Quince gel (EM+QG), and endometriosis+quince gel+hesperidin (EM+QG+HES) groups are shown in Table 1. TAS was increased and TOS was decreased significantly in the EM group compared to the sham group (*p* < 0.005). QG treatment improved scores of TAS and TOS in the EM+QG group compared to the EM group (*p* < 0.005). HES treatment after EM induction significantly increased the TAS value, and significantly decreased the TOS value in the EM+QG+HES group compared to the EM group.

### 2.2. Morphometric Findings

The morphometric measurements of the uterine tissue by groups are shown in Table 2. Uterine epithelial thickness decreased significantly in the EM group compared to the sham group. QG and HES treatment increased the thickness of uterine epithelium compared to the EM group. The diameter of the uterine glands, blood vessel lumen length, cell degeneration and inflammation scores were statistically increased in the EM group compared to the sham group. These scores were decreased in the EM+QG and EM+QG+HES groups compared to the EM group.

### 2.3. Histopathological Findings

**Figure 1:**
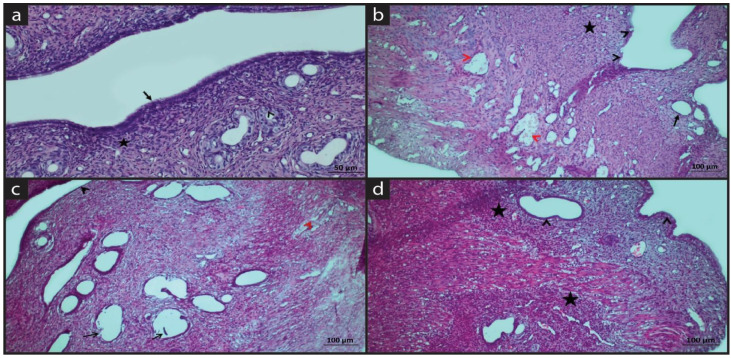
Hematoxylin-eosin staining. (**a**) Sham group: Regular epithelium (black arrow), connective tissue cells (star), normal epithelium of tubular glands (arrowhead); (**b**) EM group: Epithelial thinning, desquamation, vacuolization (black arrowhead), inflammatory cells in the lamina propria (black star), thinning of the glandular epithelium (black arrow), and vascular dilatation and thrombosis (red arrowhead); (**c**) EM+QG group: Epithelium (black arrowhead), degenerated glands (black arrow), decreased vascular dilatation and thrombosis (red arrowhead); (**d**) EM+QG+HES group: Normal epithelium and glands (black arrowhead), inflammation (black star).

Figure 1 shows hematoxylin eosin staining of experimental groups.

In the sham group, lining epithelium and glands were regular with columnar epithelium. The cilia structures were evident. The basement membrane thickness is normal. Connective tissue cells were observed in lamina propria. Vascular structures were normal in histological appearance (Figure 1a).

In the EM group, desquamation, degeneration alterations, vacuoles and thinning in epithelium were observed. Intense inflammatory cell infiltration was seen in the endometrium. Degenerative glands with thinning basement membranes were recorded. Intense dilatation and thrombosis were in vessels. Edema was present between the muscle layers. EM disrupted the histological structure and integrity of the uterus (Figure 1b).

In the EM+QG group, the uterine simple epithelium was preserved without desquamation. Degenerated glands with vacuoles were observed. Inflammatory cell infiltrations were observed in the functional and basalis layer. Vascular dilatation and thrombosis decreased. (Figure 1c).

In the EM+QG+HES group, the epithelial and glandular structure was preserved. Blood vessels were normal with mild thrombosis. Inflammatory cell infiltrations were observed near the glands and the vessels. HES with its antioxidant properties could be protective for epithelium and glands (Figure 1d).

### 2.4. Immune Staining Findings

**Figure 2:**
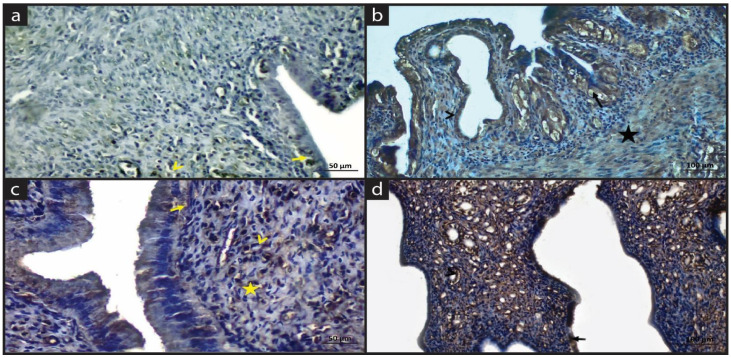
Immune staining of Caspase-3. (**a**) Sham group: Positive Caspase-3 reaction in a few cells of epithelium (arrow) and macrophages (arrowhead). (**b**) EM group: Positive Caspase-3 reaction in pyknotic nuclei of lining (black arrow) and glandular (arrowhead) epithelium, hypertrophic muscle cells (star); (**c**) EM+QG group: Positive Caspase-3 expression in cilia (arrow), macrophages (star) and plasma cells (arrowhead); (**d**) EM+QG+HES group: Positive Caspase-3 expression in epithelium (black arrow), in glands (arrowhead).

Figure 2 and Table 3 shows the Caspase-3 immune staining of experimental groups.

In the Sham group, the Caspase 3 positive reaction was observed in a few cells of the epithelium and macrophage cells. Caspase-3 expression was generally negative in the uterine (Figure 2a). In the EM group, apoptosis was increased. Caspase-3 reaction was positive in pyknotic nuclei of glands, and hypertrophic muscular cells (Figure 2b). In the EM+QG group, a negative Caspase-3 expression was observed in macrophages and plasma cells. The expression was mainly negative in epithelium, glands, and connective tissue (Figure 2c). In the EM+QG+HES group, the Caspase-3 reaction was positive in epithelial, glands and connective tissue cells (Figure 2d).

**Figure 3:**
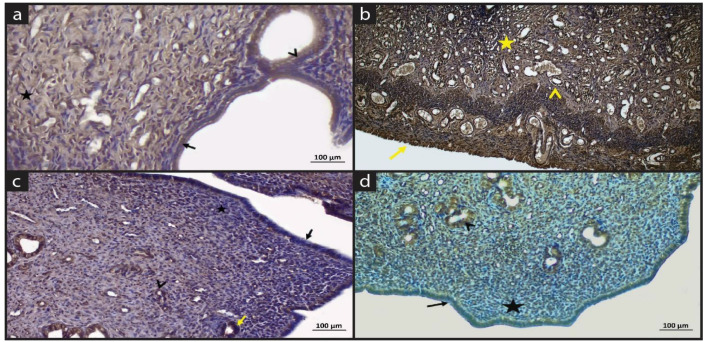
Immune staining of TNF-α. (**a**) Sham group: Positive expression in lining epithelium (black arrow), negative expression in connective tissue cells (star) and uterine glands (arrowhead); (**b**) EM group: Intense TNF-α expression in the lining epithelium (yellow arrow), glandular epithelium (yellow arrowhead), muscle layer (yellow star); (**c**) EM+QG group: Negative expression in lining epithelium (black arrow), lamina propria (star), and positive immune reaction in vessels (arrowhead) and few glands (yellow arrow); (**d**) EM+QG+HES group: Negative TNF-α expression in the epithelium (black arrow) and lamina propria (star) and positive TNF-α expression in the glandular epithelium (arrowhead).

Figure 3 and Table 4 shows the TNF-α immune staining of experimental groups. In the sham group, few nuclei of the lining epithelial cells showed positive expression, however the expression was generally negative in the lining and glandular epithelium and connective tissue cells (Figure 3a).

In the EM group, the intensity of TNF-α expression increased especially in glands, inflammatory cells, and the muscles (Figure 3b). EM+QG group mainly showed negative TNF-α immunoreactivity in the lining epithelial and glandular cells, the lamina propria (Figure 3c). In the EM+QG+HES group, the TNF-α expression was negative in the lining epithelium and lamina propria region. The TNF-α expression was positive in inflammatory cells. In general, inflammation in the EM+QG+HES group decreased (Figure 3d).

**Figure 4:**
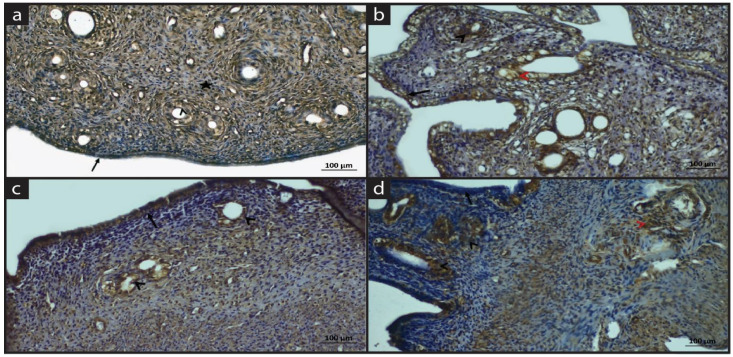
Immune staining of MAPK (**a**) Sham group: Positive MAPK expression in surface epithelium (arrow), glands (arrowhead) and macrophages (asterisk); (**b**) EM group: Positive MAPK expression in the epithelium (black arrow), vessels (black arrowhead), glands (red arrowhead) in the EM group; (**c**) EM+QG group: Positive MAPK expression in the epithelium (black arrow), uterine glands (black arrowhead); (**d**) EM+QG+HES group: Negative MAPK expression in the epithelium and lamina propria (black arrow), positive MAPK expression in glands and inflammatory cells (black arrowhead), vessel (red arrowhead).

Figure 4 and Table 5 shows the MAPK immune staining of experimental groups. In the sham group, the MAPK immune reaction was positive in the lining epithelium, glands and endothelial cells. In general, cells in the lamina propria showed negative MAPK immune reactions; however, macrophages showed positive MAPK expression (Figure 4a).

In the EM group, the MAPK expression was positive in epithelium, glandular cells, connective tissue cells, inflammatory cells and vessels. It can be stated that MAPK initiates the signaling pathways in the endometriosis group (Figure 4b).

In the EM+QG group, the MAPK expression was positive in epithelial cells. Moderate MAPK expression was observed in connective tissue cells. Quince gel shows a tissue protective effect and downregulates the signaling (Figure 4c).

In the EM+QG+HES group, the MAPK expression was positive lining epithelium, inflammatory cells and endothelial cells while the expression was negative in the lamina propria. While quince gel alone restored the pathology and downregulated the MAPK signaling, the combination of HES with quince gel has an especially anti-inflammatory effect (Figure 4d).

### 2.5. Ultrastructural Findings

**Figure 5:**
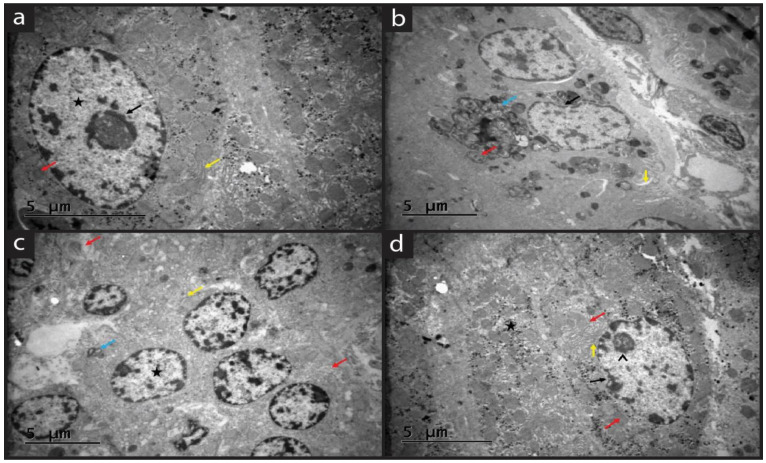
Electron microscope graphs. (**a**) Sham group: Nucleolus rich chromatin (black arrow), euchromatin (star), normal endoplasmic reticulum (yellow arrow) and mitochondria (red arrow); (**b**) EM group: Fragmented hyalinized areas (blue arrow), notched nucleus appearance (black arrow), dilatation of endoplasmic reticulum (yellow arrow), loss of cristae in mitochondria (red arrow); (**c**) EM+QG group: Round nucleus (star), dilatation of endoplasmic reticulum (yellow arrow), normal mitochondria (red arrow), terminal web (blue arrow); (**d**) EM+QG+HES group: Increased inclusion (star), notched nucleus (black arrow), normal endoplasmic reticulum (yellow arrow), electron-dense mitochondria (red arrow), nucleolus (arrowhead).

Figure 5 shows the ultrastructural images of uterine epithelial cells by experimental groups. In the sham group, the nucleus was rich in chromatin with euchromatin. Crista of mitochondrial were evident. The endoplasmic reticulum was located near the nucleus with cisterns. Tubular endoplasmic cisterns are located near the luminal surface of the cytoplasm. Few inclusion bodies were seen. The Golgi apparatus was prominent (Figure 5a).

In the EM group, the nuclei were round with euchromatic. There was organelle degeneration in the cytoplasm, especially mitochondria and lysosomes. The loss of cristae in mitochondria was evident. There were fragmented and hyalinized areas in the cytoplasm. Slight enlargement and degeneration were observed in the endoplasmic reticulum cisterns. Loss of cytoplasm was observed with a shrunk nucleus which showed degenerated cells (Figure 5b).

In the EM+QG group, although there was a partially shrunken nucleus, normal round nuclei and heterochromatin features were observed. In general, the nucleus structure was preserved. Mitochondrial cristae were normal. Mild dilated ER was seen. No abnormality was observed in this group in general. The terminal web was prominent (Figure 5c).

There was an increase in inclusion bodies in the EM+QG+HES group. Electron-dense mitochondria were present. Normal endoplasmic reticulum was seen with non-dilated cisterna appearance. The combination of QG+HES provided the protection of the endoplasmic reticulum. A significant reduction in the nucleolus volume was observed. Mitochondria appeared normal (Figure 5d).

## 3. Discussion

Endometriosis (EM) is the presence of endometrial tissue outside the uterine cavity. Many hypotheses have been proposed for the etiology of endometriosis; however, the exact mechanism is unknown. The most accepted theory is retrograde menstruation. There is no definitive treatment for endometriosis [26]. For centuries, medicinal plants have been used in the treatment of various diseases. Hesperidin (HES) is a natural bioflavonoid found in citrus fruits such as oranges, lemons, and tangerines with various biological activities [27]. Beskisiz et al. [14] investigated the role of hesperidin in immobilization stress in rats. The authors noted that the antioxidant property of hesperidin improves gastric mucosa in gastric lesions that develop as a result of stress, and decreases the oxidant markers malondialdehyde (MDA) and myeloperoxidase (MPO) values to normal levels after stress. Li et al. [28] investigated the antioxidant properties of hesperidin in their study on hepatic ischemia-reperfusion injury and emphasized that hesperidin treatment after ischemia reperfusion inhibited inflammation signaling pathways, prevented hepatocyte apoptosis, promoted cell survival with the AKT signaling pathway, and protected hepatic tissue. The protective effects of hesperidin due to its antioxidant properties were investigated in a study by Çelik et al. [29]. The authors noted that after torsion detorsion, hesperidin treatment decreased malondialdehyde (MDA) levels and increased the activities of antioxidant molecules superoxide dismutase (SOD), catalase (CAT) and glutathione (GSH). In this study, total antioxidant status (TAS) was increased and total oxidant status (TOS) was decreased significantly in the EM group. HES with quince gel (QG) given after EM significantly increased the TAS content and decreased the TOS content in the EM+QG+HES group compared to the EM group.

Endometriosis changes the histology of uterine tissue. Kennedy et al. [30] stated that the morphology of endometriosis is superficial “powder burn” or “gunshot” lesions; black, dark-brown, or bluish puckered lesions, nodules, or small cysts with hemorrhage and increased fibrosis. Mashele et al. [31] showed that endometriosis causes pathology in tissues such as topographic sites, endometrial glands, hemorrhage, endometriotic cysts, and ciliated metaplasia. These pathologies were recorded in the fallopian tubes, the umbilical region, and the abdominal wall. In the present study, EM induction caused degenerative epithelial cells, increased inflammatory cells, thinning of the basement membrane, vascular dilatation and thrombosis. QG and HES treatment restores the histopathology of uterine tissue after EM induction. HES protected the uterine tissue with its antioxidant and anti-inflammatory activity.

Caspases are cysteine proteases that are involved in extrinsic and intrinsic pathways of apoptosis and inflammatory responses. They cleave various substrates in the apoptotic cells [32]. Caspase-3 belongs to the effector caspase group and causes apoptotic cell morphology by degrading the relevant proteins in the cell that will undergo apoptosis [33]. Li et al. [34] reported that Caspase-3 played the most important role in the apoptotic process and showed similar properties to caspase-9. Kim et al. [35] studied cardiac ischemia-reperfusion and showed that oligonucleosomal DNA fragments were formed with the activation of Caspase-3 and the cell entered an irreversible pathway with the appearance of apoptotic bodies. Kaya et al. [36] studied the serum level of Caspase-3 in endometrioma patients and found that the Caspase-3 level was higher in patients with stage I–II and stage III–IV endometrioma compared to the sham group. They claim that the Caspase-3 serum level expression can be a good predictor of the severity of endometriosis. In the present study, EM led to the increased expression of Caspase-3 in epithelial, glandular, and inflammatory cells. QG and HES treatment decreased the Caspase-3 expression by their cell survival activity in EM+QG and EM+QG+HES groups.

Tumor necrosis factor-α (TNF-α) is a cytokine produced by various immune cells that include macrophages/monocytes. TNF-α can induce multiple signal pathways involved in inflammation, proliferation, and apoptosis. TNF-α has long been reported as an important tool in the diagnosis of endometriosis [37,38]. Galo et al. [39] studied TNF-α serum level as a prediction of endometriosis in 65 women. They measured the TNF-α level and found that the average serum level of TNF-α in patients with endometriosis was higher than in patients without endometriosis. The authors claim that TNF-α level can be a good predictor of endometriosis in non-invasive methods. Lu et al. [40] investigated the anti-TNF-α treatment for pelvic pain in premenopausal patients with endometriosis. They suggested that there is still not enough evidence to support TNF-α treatment for pelvic pain in patients with endometriosis. In the present study, the EM group showed a high level of TNF-α immune reaction in epithelial, glandular and inflammatory cells. QG and HES treatment decreased the TNF-alpha expression with their anti-inflammatory effects in the EM+QG and EM+QG+HES groups.

Mitogen-activated protein kinases (MAPKs) are highly conserved motifs between eukaryotes and one of the largest groups of transferases. They are serine and threonine protein kinases [41]. MAPK signaling pathway performs many cellular functions such as proliferation, differentiation, apoptosis, inflammation, and the regulation of the immune system. Any disruption in this signaling pathway causes healthy cells to turn into cancer or cause problems at the developmental stage. MAPKs respond to various extracellular signals by direct phosphorylation of the kinase at the top of the pathway, activating the middle kinase and then activation of the last kinase [42,43]. This study showed that EM induction caused elevated MAPK immunoreactivity in the lamina propria, glands, and inflammatory cells. In the EM+QG group, the MAPK expression was positive in epithelial cells and gland cells. In the EM+QG+HES group, the MAPK expression was positive in the epithelium, the inflammatory cells, and vessels.

Ultrastructural analysis of the epithelial cells of uterine tissue showed that in the sham group, organelles of uterine cells were normal. In the EM group, degenerated nuclei and cytoplasm, with a disruption in mitochondria, ER, and Golgi apparatus. Organelles were dilated. In the EM+QG and EM+QG+HES group, the nucleus structure was preserved. Mitochondrial cristae were normal with regular mitochondria. QG+HES treatment protected the uterine cells ultrastructurally.

In the molecular mechanism of the pathophysiology of EM, apart from implementation, many factors may have impacts on it [44]. Hormones, cytokines, growth factors, and other factors present in peritoneal or ovarian fluid or the bloodstream may have a role in EM development [45]. TNF-α is known to be a proliferative factor for EM [46,47]. In this study, we showed that Caspase-3, TNF-α, and MAPK expression were increased in EM. Increased TNF-α expression activated apoptotic evasion and led to increased expression of Caspase-3 and MAPK. We suggest that the Caspase-3, and MAPK with TNF-α may involve in the pathophysiology of EM.

## 4. Materials and Methods

### 4.1. Plant Sampling and Gel Preparation

Fresh quince fruit was purchased from a local market (Diyarbakır, Turkey). Seeds were extracted from the fruit. A total of 10 quince seeds were soaked in 10 cc of distilled water overnight to obtain the gel form of quince. This procedure was repeated daily to obtain fresh quince gel (QG). The active ingredient hesperidin (catalog no: H5254) was purchased commercially from Sigma Aldrich (Hamburg, Germany).

### 4.2. Experimental Design

Animal experiments were conducted in accordance with ethical approval from Dicle University, Animal Experiments Local Ethical Committee (date: 24 February 2022 and no: 2021/42). All experimental interventions were under general anesthesia with 90 mg/kg intramuscular ketamine hydrochloride (Ketalar, Pfizer) and 8 mg/kg xylazine (Rompun, Bayer). The experimental endometriosis model was created according to the study of Vernon and Wilson et al. [48]. The abdominal cavity was dissected and uterine horns were observed. Then, a small portion of the left uterine horn was excised and implanted on the right peritoneal surface. The abdominal cavity was sutured, and animals were placed back into their cages. After 4 weeks, animals with endometriosis were included for further interventions. To create endometriosis hyperplasia, estradiol benzoate (EB) was dissolved in 5% corn oil. In total, 32 Sprague Dawley male rats (12-weeks old, 200–250 gr) were categorized into four groups (8 rats per group). Experiments were imitated after the estrus stages of female rats were determined.

1. Sham group (*n* = 8): Animals underwent operation without any further intervention and no endometrial implantation. Then rats were housed in cages for 7 days and sacrificed at the end of the 7th day.
2. Endometriosis (EM) group (*n* = 8): 3 µg/kg EB was administered subcutaneously to the rats for 7 days. Animals were sacrificed at the end of the 7th day.
3. Endometriosis+Quince gel (EM+QG) group (*n* = 8): 3 µg/kg EB was administered subcutaneously to the rats for 7 days. Concurrently with EB administration, 2cc of QG was applied to the vagina with a thin catheter daily for 7 days and additionally for 14 days (totally 21 days). Animals were sacrificed at the end of the 21st day.
4. Endometriosis+Quince gel+hesperidin (EM+QG+HES) group (*n* = 8): After the estrus stages of female rats were determined, 3 µg/kg EB was administered subcutaneously to the rats for 7 days. Concurrently with EB administration, 50 mg/kg/day Hesperidin with 2cc of QG was applied to the vagina with a thin daily for 7 days and additionally for 14 days (total 21 days). Animals were sacrificed at the end of the 21st day.

### 4.3. Biochemical Analysis

Intracardiac blood was processed for biochemical analysis according to Durgun et al. [49]. Blood was centrifugated at 12,000 rpm for 5 min and serum was used in assays to measure total antioxidant status (TAS) and total oxidant status (TOS). Contents were measured spectrophotometrically using a biochemical autoanalyzer (AU5800; Beckman Coulter, Inc., Brea, CA, USA). TAS values were recorded as μmol H_2_O_2_ Eq/L and TOS values were recorded as μmol Trolox Eq/L.

### 4.4. Histological Follow-Up

The abdomen of the rats was opened, and the uterine tissue was dissected. Uterine samples were fixed in 10% formalin for 24 h. Tissues were dehydrated with increased grading alcohol series and incubated in paraffin infiltration. Then, the tissues were embedded in paraffin blocks and 5 μm thick sections were stained for Hematoxylin-Eosin and immunoreaction.

### 4.5. Histological Staining

Uterine sections were deparaffinized and passed through decreased alcohol grading series and brought to distilled water. Hematoxylin eosin stainings were applied to the sections. Sections were quickly immersed in increasing alcohol series (through 80%, 90%, 96%, absolute alcohol) and cleared in xylene for 3 × 15 min. Sections were mounted and analyzed A histological scoring was completed to measure cell degeneration and inflammation. Ten areas for each animal were analyzed by two blind experts for cell degeneration and inflammation. Scoring was calculated as 1: none, 2: mild, 3: moderate, 4: severe [50].

### 4.6. Immunohistochemical Staining

Uterine tissue sections were deparaffinized, dehydrated, and washed in distilled water. Hydrogen peroxide solution (cat# TA-015-HP, ThermoFischer, Fremont, CA, USA) was dropped onto the sections and incubated for 20 min. Sections were kept in Ultra V Block (cat#TA-015-UB, ThermoFischer, USA) solution for 7 min. Sections were washed with primary antibodies caspase-3 (cat# A60144), TNF-α (cat# A42662), MAPK (cat#A46000) (AFG Bioscientific, US, dilution ratio:1/100) overnight at +4 °C. sections were incubated with biotinylated secondary antibody (cat#TP-015-BN, ThermoFischer, USA) for 14 min. Streptavidin-peroxidase (cat#TS-015-HR, ThermoFischer, USA) was reacted with sections for 15 min. Diaminobenzidine (DAB) (cat#TA-001-HCX, ThermoFischer, USA) was used for chromogen to observe protein expression. Sections were washed with PBS and counterstained with Harris hematoxylin. Slides were mounted and analyzed by Zeiss Imager A2 light microscope.

### 4.7. Ultrastructural Tissue Preparation

Uterine samples were dissected and fixed in 4% buffered glutaraldehyde and then, in 1% osmium tetroxide for transmission electron microscopic tissue preparation. Uterine sections were placed on copper grids and counterstained with uranyl acetate-lead citrate. The grids were evaluated in Jeol 1010 transmission electron microscope for imaging.

### 4.8. ImageJ Analysis

The staining intensity of Caspase-3, TNF-a, and MAPK expression was measured by Image J software (version 1.53, http://imagej.nih.gov/ij). Measurement was calculated by the method of Crowe et al. [51]. For each sample, five sections with ten areas were analyzed per group.

### 4.9. Statistical Analysis

All statistical analyses were performed with the IBM SPSS Statistics version 25 software program. Data distribution was made using the Kolmogorov–Smirnov test. Comparisons between groups were made with Kruskal–Wallis and post hoc Dunn’s test. Data were recorded as median (min–max). A *p*-value of <0.05 was considered statistically significant.

## 5. Conclusions

Endometriosis promoted acceleration of vasculogenesis and inflammation with the apoptotic pathway by inducing Caspase-3 and TNF-α and MAPK signaling pathways in endometriotic cells. Quince gel inhibited inflammation by stimulating the cell proliferation process and the MAPK pathway. Inflammation and apoptotic process begin to slow down due to the high antioxidative and anti-inflammatory effect of the mixture of quince gel and hesperidin which led to increased euchromatin nucleus and reduced mitochondrial damage in uterine cells. In this endometriosis model, vaginal local treatment was thought to be an important treatment method in restoring the uterine mucosa via the promotion of cell proliferation, angiogenesis and prevention of inflammation.

## Figures and Tables

**Table 1:** Comparison of TAS and TOS values between groups.

Groups	TAS	TOS	*p*
Sham	1.45 (0.76–1.65)	15.73 (10.34–55.18)	<0.005
EM	0.95 (0.58–1.11) ^a^	35.80 (21.76–88.85) ^a^	
EM+QG	1.09 (0.66–1.21) ^b^	29.92 (19.48–57.91) ^b^	
EM+QG+HES	1.21 (0.92–1.47) ^c^	21.42 (16.13–33.47) ^c^	

EM: endometriosis, QG: quince gel; HES: hesperidin, TAS: total antioxidant status, TOS: total oxidant status, a: EM vs. Sham; b: vs. EM; c: vs. EM.

**Table 2:** Distribution of morphometric parameters by groups.

Parameters	Sham Median (min-max)	EM Median (min-max)	EM+QG Median (min-max)	EM+QG+HES Median (min-max)
Uterine epithelial thickness (μm)	23.45 (16.45–29.45)	8.05 (6.88–8.92) *	14.03 (10.91–18.98) **	16.1135 (11.58–19.99) **
Diameter of uterine glands (μm^2^)	973.45 (956.45–1023.35)	1697.51 (1678.71–1714.07) *	1386.48 (1027.78–1909.82) **	1281.85 (958.68–1891.53) **
Blood vessel lumen length (μm)	77.68 (66.34–84.57)	97.95 (86.30–106.11) *	92.63 (85.06–104.66) **	86.62 (71.11–99.35) **
Cell degeneration	1 (1–2)	3 (3–4) *	3 (2–4) **	3 (1–4) **
Inflammation	1 (1–2)	4 (3–4) *	3 (2–4) **	3 (1–4) **

* sham vs. EM (*p* < 0.05); ** EM vs. treatment groups (*p* < 0.05).

**Table 3:** Image J analysis of immunohistochemistry per group.

Groups	Sham	EM	EM+QG	EM+QG+HES
Caspase-3	%15.21	%47.44	%40.86	%38.28

Note: percentage shows ratio of signal intensity to whole area of a section.

**Table 4:** Image J analysis of immunohistochemistry per group.

Groups	Sham	EM	EM+QG	EM+QG+HES
TNF-α	%18.35	%43.92	%39.02	%35.18

Note: percentage shows ratio of signal intensity to whole area of a section.

**Table 5:** Image J analysis of immunohistochemistry per group.

Groups	Sham	EM	EM+QG	EM+QG+HES
MAPK	%22.45	%37.67	%27.83	%30.56

Note: percentage shows ratio of signal intensity to whole area of a section.

## Data Availability

All generated data were presented in this study.
